# Excess Mortality during the COVID-19 Pandemic in Cities of Chile: Magnitude, Inequalities, and Urban Determinants

**DOI:** 10.1007/s11524-022-00658-y

**Published:** 2022-06-10

**Authors:** Tania Alfaro, Kevin Martinez-Folgar, Alejandra Vives, Usama Bilal

**Affiliations:** 1 Escuela de Salud Pública, Facultad de Medicina, Universidad de Chile, Independencia 939, Santiago, Chile; 2 Urban Health Collaborative; and Department of Epidemiology and Biostatistics, Drexel Dornsife School of Public Health, Philadelphia, PA USA; 3 Departamento de Salud Pública, Pontificia Universidad Católica de Chile, CEDEUS, Santiago, Chile

**Keywords:** COVID-19, Mortality, Chile, Urban health

## Abstract

We estimated excess mortality in Chilean cities during the COVID-19 pandemic and its association with city-level factors. We used mortality, and social and built environment data from the SALURBAL study for 21 Chilean cities, composed of 81 municipalities or “*comunas*”, grouped in 4 macroregions. We estimated excess mortality by comparing deaths from January 2020 up to June 2021 vs 2016–2019, using a generalized additive model. We estimated a total of 21,699 (95%CI 21,693 to 21,704) excess deaths across the 21 cities. Overall relative excess mortality was highest in the Metropolitan (Santiago) and the North regions (28.9% and 22.2%, respectively), followed by the South and Center regions (17.6% and 14.1%). At the city-level, the highest relative excess mortality was found in the Northern cities of Calama and Iquique (around 40%). Cities with higher residential overcrowding had higher excess mortality. In Santiago, capital of Chile, municipalities with higher educational attainment had lower relative excess mortality. These results provide insight into the heterogeneous impact of COVID-19 in Chile, which has served as a magnifier of preexisting urban health inequalities, exhibiting different impacts between and within cities. Delving into these findings could help prioritize strategies addressed to prevent deaths in more vulnerable communities.

**Supplementary Information:**

The online version contains supplementary material available at 10.1007/s11524-022-00658-y.

## Introduction

The COVID pandemic had caused more than 5 million confirmed deaths worldwide as of October 2021 [1]. However, the total mortality burden of COVID-19 may exceed this number, as there are heterogeneous criteria used to attribute deaths to COVID-19, together with the limited coverage of testing in many countries [2]. Moreover, strategies to mitigate the COVID-19 pandemic may themselves generate increases or decreases in mortality, beyond their direct effects on COVID-19 transmission. For example, social distancing may result in reductions in mortality due to injuries and delays in seeking health care potentially impacting mortality by noncommunicable diseases [2, 3]. These limitations make it challenging to compare the scale of the pandemic between and within countries. To address this limitation, excess mortality (EM) is an indicator which has been used to evaluate the magnitude and severity of different pandemics like influenza and other catastrophic events like wars or humanitarian emergencies [4–6]. Estimating excess deaths could be a more accurate measure of the impact of COVID-19, including deaths directly or indirectly caused or even prevented by the pandemic through the comparison of current mortality patterns with that of previous years [7, 8].

During the pandemic, various countries have reported heterogeneous impacts of COVID-19 across their territory [9–12]. Potential reasons for these include the role of environmental factors on COVID-19 transmission and severity [13–15]. For example, although different studies described a decrease in the air pollution levels during the pandemic, and as well in Chile [16], other have related higher levels of PM2.5 with higher incidence, hospital admissions and mortality by COVID-19, suggesting an increase in the disease`s severity related with air pollution [13–15]. Population density has showed a correlation with morbidity and mortality [17], although there are some contradictory results [18]. Studies have shown differences in EM across age, sex and educational level [10, 19, 20], while others showed lower mortality rates in counties and cities with better socioeconomic indicators [18, 21–23].

Here, we aim to describe the EM during the COVID-19 pandemic in 2020 and the first half of 2021 in 21 Chilean cities and their corresponding 81 municipalities, and to explore associations with city and municipality-level social and built environment factors. While most descriptions of EM have focused on countries in the Global North [24–26], we focus on Chile, a high-income country with wide health inequalities [27–29], in COVID-19 incidence and mortality too [23, 30], and a well-developed mortality registration system that has been releasing almost real time counts nationally and for each municipality. Moreover, we focus on cities beyond the capital (Santiago), where most of the research on health inequities has been conducted so far [23, 30].

## Methods

This analysis was conducted as part of the SALURBAL study, which collects data on health and the environment in cities of 11 Latin American countries [31, 32], defining cities as agglomerations of municipalities that overlap with the urban extent of a city or urban area with more than 100,000 residents by 2010 [31, 32]. Here, we focused on the 21 cities and their corresponding 81 municipalities for which data is available in SALURBAL in Chile [31, 32], out of their 346 municipalities, representing 23% of Chilean municipalities but 70% of the total population. Cities were grouped by macroregion, defined by the Ministry of Social Development [33] (North, Center, Metropolitan, and South region, after regrouping the South and extreme South regions).

We obtained mortality data from January 2016 to June 2021 by age, sex and municipality from the vital registration system of Chile (Department of Statistics and Health Information) [34].We obtained population denominators for the same period and social and built environment variables from the SALURBAL study. Details on the sources of these data are described elsewhere [31, 32]. To account for potential delays in mortality statistics, we used data on deaths registered up to September 1^st^ 2021, covering the period of deaths occurring from January 1^st^ 2016 to July 3^rd^, 2021 (26^th^ epidemiological week, 2021).

To characterize the social environment of each city and municipality we used four indicators: two for educational attainment (% of the population aged 25 or above with completed university education, or with completed secondary education), poverty (% of the population that cannot fulfill their basic needs), and overcrowding (% of households with 2.5 or more people per bedroom). Educational attainment and poverty are used as indicators for area-level socioeconomic status, which has been found to be predictive of COVID-19 transmission and mortality in several settings [23, 35, 36]. Overcrowding was selected as it represents one of the key drivers of COVID-19 transmission [35]. All were computed at the city and municipality level and obtained from the 2015 National Socio-Economic Characterization Survey (CASEN) for poverty, and from the 2017 Chilean Census for all other indicators.

To characterize the built and physical environment of each city and municipality, we used data on population density (people per km^2^, 2020) and air pollution (annual mean of population-weighted PM2.5, 2018) [37, 38], considering the evidence which associates PM2.5 concentration with COVID-19 severity [39]. We also used data on total city size for analysis at the city-level. Details on these data sources are available elsewhere [31, 40].

### Statistical Analysis

The main objectives were to estimate EM from January 2020 to June 2021 in 21 Chilean cities and their respective 81 municipalities, and to explore associations between city- and municipality-level social and built environment indicators and EM. We conducted this analysis in three steps. First, we described average monthly deaths by period (2016–2019, 2020, and 2021) overall, for SALURBAL Chilean cities (considering the 81 municipalities), and by sex and age groups (< 5, 5 to 19, 20 to 39, 40 to 59, 60 to 74 and 75 +).

Second, we estimated weekly and total (sum for the whole period) EM (and 95% confidence intervals (CI)) in cities pooled by four macroregion, each city, and each municipality. We calculated EM by computing a smoothed estimate of expected death counts for each week of 2020 and up to June 2021 based on data from 2016 to 2019, using a negative binomial generalized additive model (GAM), as detailed in Basellini et al [41], with a population offset (more details in supplementary material 2). This model takes into consideration both variations in mortality within the year and secular trends. We used it to compute weekly EM and calculated total EM during 2020 up to June 2021, and associated 95% CI. We conducted two sensitivity analyses to assess the robustness of our EM estimates. First, we tested three different baseline windows: using data from 2016 to 2019 (main analysis), 2017 to 2019, and 2018 to 2019. Second, we compared our estimates of EM as obtained from the GAM with an empirical estimate obtained by comparing deaths in each week of 2020 or 2021 with the average of deaths in the same week during the 2016–2019 period.

Absolute EM was defined as the difference between observed deaths and expected deaths (obtained from the GAM model) divided over the population in 2020 and 2021, and relative EM was defined as EM over the number of expected deaths. We show these results in three ways: overall, summing over all 81 municipalities of Chile belonging to SALURBAL; by city and municipality; by the 4 macroregions.

Third, we studied the associations between social and built environment factors and EM at the ecological level at two different levels. We ran a linear model at the city-level to estimate the strength of associations between these factors and relative and absolute EM in January 2020-June 2021, adjusting for age (% of the population aged 65 or above). We repeated the same process for the municipalities belonging to three large metropolitan areas in Chile: the conurbation of Santiago in the Metropolitan Region, Valparaíso in the Valparaíso Region and Concepción in the Biobío Region. We selected these areas as they are the only ones with five or more sub-units, allowing for an exploration of within-city differences. In a secondary analysis, and to acknowledge uncertainty around the estimation of EM, we used the same procedure as Basellini et al [41] (supplementary material 2).

All the analyses were performed in R v4.1. The GAM was estimated using the mgcv package.

## Results

**Fig. 1:**
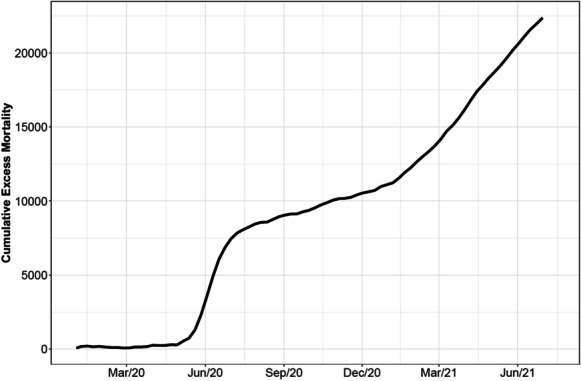
Cumulative mortality during the COVID-19 pandemic in 21 Chilean cities (January 2020-June 2021)

Table 1 shows average number of monthly deaths for the 2016–2019, 2020 and 2021 periods, overall, for SALURBAL cities, sex, and age (supplementary material, Figs. 1 through 3, show weekly rates). The all-cause mortality rate in Chile in 2016–2019, 2020, and 2021 was 573, 647, and 636 deaths per 100,000, representing a 13 and 11% increase in 2020 and 2021 (up to July 3^rd^), respectively. Increases were especially salient in men and older adults, with similar patterns in the whole country compared to SALURBAL cities. Figure 1 displays the cumulative EM between January 2020 to June 2021. By the end of epidemiological week 26, 2021, the 21 Chilean cities experienced a total of 21,699 excess deaths (95%CI 21,693 to 21,704), with around half occurring in 2020.

**Table 1:** Average monthly deaths, overall, and by sex, and age, in Chile, January 2016–June 2021

		Period	Monthly deaths (thousands)												Population (millions)
			Jan	Feb	Mar	Apr	May	Jun	Jul	Aug	Sep	Oct	Nov	Dec	
	Overall	2016–2019	8.27	7.51	7.99	8.26	9.14	10.16	10.60	9.96	9.33	8.87	8.25	8.26	18.6
		2020	9.05	7.94	8.70	8.89	11.99	16.04	12.58	11.08	10.21	10.32	9.37	9.68	19.5
		2021	11.20	10.24	11.85	11.98	12.76	13.52							19.7
	SALURBAL cities	2016–2019	5.67	5.15	5.51	5.68	6.30	7.00	7.34	6.87	6.39	6.05	5.62	5.68	13.2
		2020	6.21	5.42	5.93	6.13	8.84	12.17	8.92	7.73	7.00	7.04	6.32	6.50	13.9
		2021	7.57	6.89	8.02	8.29	8.72	9.30							14.0
Sex	Male	2016–2019	4.40	3.99	4.24	4.36	4.83	5.31	5.51	5.12	4.88	4.64	4.33	4.37	9.2
		2020	4.81	4.29	4.60	4.70	6.43	8.75	6.79	5.85	5.46	5.49	5.03	5.25	9.6
		2021	6.01	5.59	6.43	6.37	6.91	7.33							9.7
	Female	2016–2019	3.87	3.51	3.75	3.90	4.31	4.85	5.08	4.83	4.44	4.22	3.92	3.89	9.4
		2020	4.24	3.64	4.09	4.19	5.56	7.29	5.79	5.23	4.75	4.83	4.33	4.43	9.9
		2021	5.19	4.65	5.42	5.62	5.85	6.20							10.0
Age	< 5	2016–2019	0.16	0.13	0.15	0.14	0.15	0.16	0.16	0.15	0.14	0.15	0.13	0.14	1.2
		2020	0.12	0.10	0.11	0.11	0.11	0.12	0.11	0.11	0.11	0.11	0.08	0.09	1.2
		2021	0.11	0.11	0.11	0.09	0.11	0.10							1.2
	5–19	2016–2019	0.08	0.07	0.07	0.07	0.07	0.08	0.08	0.08	0.08	0.07	0.07	0.07	3.8
		2020	0.09	0.06	0.06	0.06	0.06	0.07	0.06	0.06	0.06	0.07	0.07	0.07	3.8
		2021	0.07	0.07	0.07	0.07	0.06	0.06							3.8
	20–39	2016–2019	0.40	0.37	0.37	0.36	0.37	0.38	0.38	0.40	0.39	0.38	0.38	0.40	5.8
		2020	0.44	0.41	0.40	0.38	0.48	0.52	0.43	0.40	0.40	0.42	0.40	0.43	6.1
		2021	0.45	0.40	0.50	0.44	0.54	0.54							6.2
	40–59	2016–2019	1.22	1.12	1.18	1.15	1.26	1.34	1.36	1.26	1.24	1.20	1.16	1.20	4.8
		2020	1.21	1.12	1.22	1.23	1.64	2.08	1.65	1.42	1.37	1.48	1.25	1.29	5.0
		2021	1.53	1.44	1.69	1.85	2.02	1.93							5.1
	60–74	2016–2019	2.15	2.01	2.17	2.20	2.40	2.59	2.69	2.54	2.41	2.32	2.16	2.11	2.2
		2020	2.37	2.09	2.39	2.33	3.35	4.75	3.58	2.98	2.73	2.86	2.56	2.61	2.4
		2021	3.06	2.82	3.40	3.48	3.52	3.65							2.5
	75 +	2016–2019	4.27	3.81	4.05	4.34	4.89	5.62	5.92	5.54	5.07	4.75	4.35	4.34	0.9
		2020	4.83	4.15	4.52	4.79	6.34	8.50	6.74	6.11	5.54	5.39	5.02	5.19	1.0
		2021	5.99	5.41	6.08	6.05	6.53	7.25							1.0

Footnote: 2016–2019 refers to the average monthly death count or yearly population from 2016 to 2019. All death counts are represented in thousands, population is represented in millions. Urban is defined as a municipality that is in a city with more than 100,000 residents

**Fig. 2:**
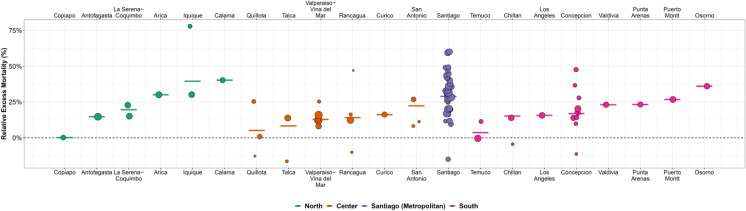
Excess mortality by municipality in 21 Chilean cities

Figure 2 shows the overall EM in the cities and municipalities studied, illustrating the heterogeneous impact COVID-19 has had across the bigger cities of Chile. Calama (North) had the highest increase in mortality at 40.3%, followed by Iquique (39.5%) in the North, and Osorno (36%) in the South. Santiago, the capital, had an EM of around 30%. Copiapó had the lowest impact around 0.2%, followed by Quillota and Temuco (lower than 6%), in the North, Center, and South regions, respectively. We also found wide heterogeneity within cities, with municipalities of Santiago seeing increases in mortality of 60% (Cerro Navia, Peñalolén), or even declines in mortality of 15% (La Reina), with similar heterogeneity in the metropolitan area of Concepción (48% in Hualpén vs 10% in Tomé). The highest EM was observed in the Municipality of Alto Hospicio in Iquique, reaching a 78% EM. Absolute EM was highest in Osorno (206 excess deaths per 100,000), followed by Arica, Iquique and Calama (between 159 and 154 excess deaths per 100,000). Within cities, the highest absolute EM was observed in San Ramón and Cerro Navia (394.8 and 386.7 excess deaths per 100,000, respectively), while the lowest was observed in La Reina (-132.5 excess deaths per 100,000). Supplementary material Figure 4 details weekly EM for each of the cities, while supplementary material Fig. 6 shows a map of the distribution of EM by city. Supplementary material Figs. 7, 8, and 9 show the results of the EM analysis using different baseline windows and doing an empirical comparison with the average weekly deaths of prior years. In the analysis comparing 2020 and 2021 with the weekly averages of deaths in 2016–2019, we found that our model produced slightly lower estimates of EM compared to the empirical approach, but that the correlation between both sets of EM estimates was high (Spearman’s rho = 0.75 and 0.73 for relative and absolute EM).

**Fig. 3:**
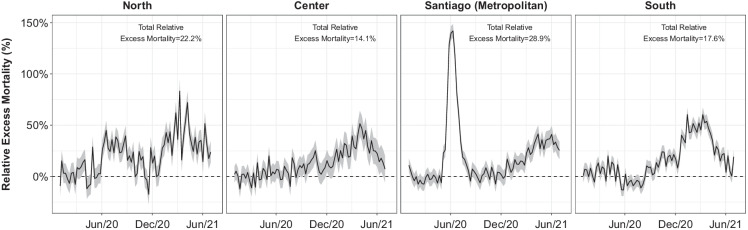
Weekly trends in excess mortality from January 2020 to June 2021 in 21 Chilean Cities by macroregion

Figure 3 shows trends in EM in 21 Chilean cities by week and macroregion. We found that Metropolitan (Santiago) and the North regions  had the highest overall EM (28.9% and 22.2%, respectively), followed by the South and Center regions (17.6% and 14.1%). Santiago had the highest peak EM at almost 150% during weeks 23 and 24 (first half of June 2020), with weeks 21 and 22 (second half of May) and 25–27 (second half of June) having increases > 50%. The other macroregions, had higher EM during 2021 than 2020, with increases > 70% during weeks 10 and 14 (beginning of March and April 2021, respectively) in the North, during week 13 (late March 2021) in the Center (around 50%) and during weeks 2 (early January) and 10 in the South (> 60%).

**Fig. 4:**
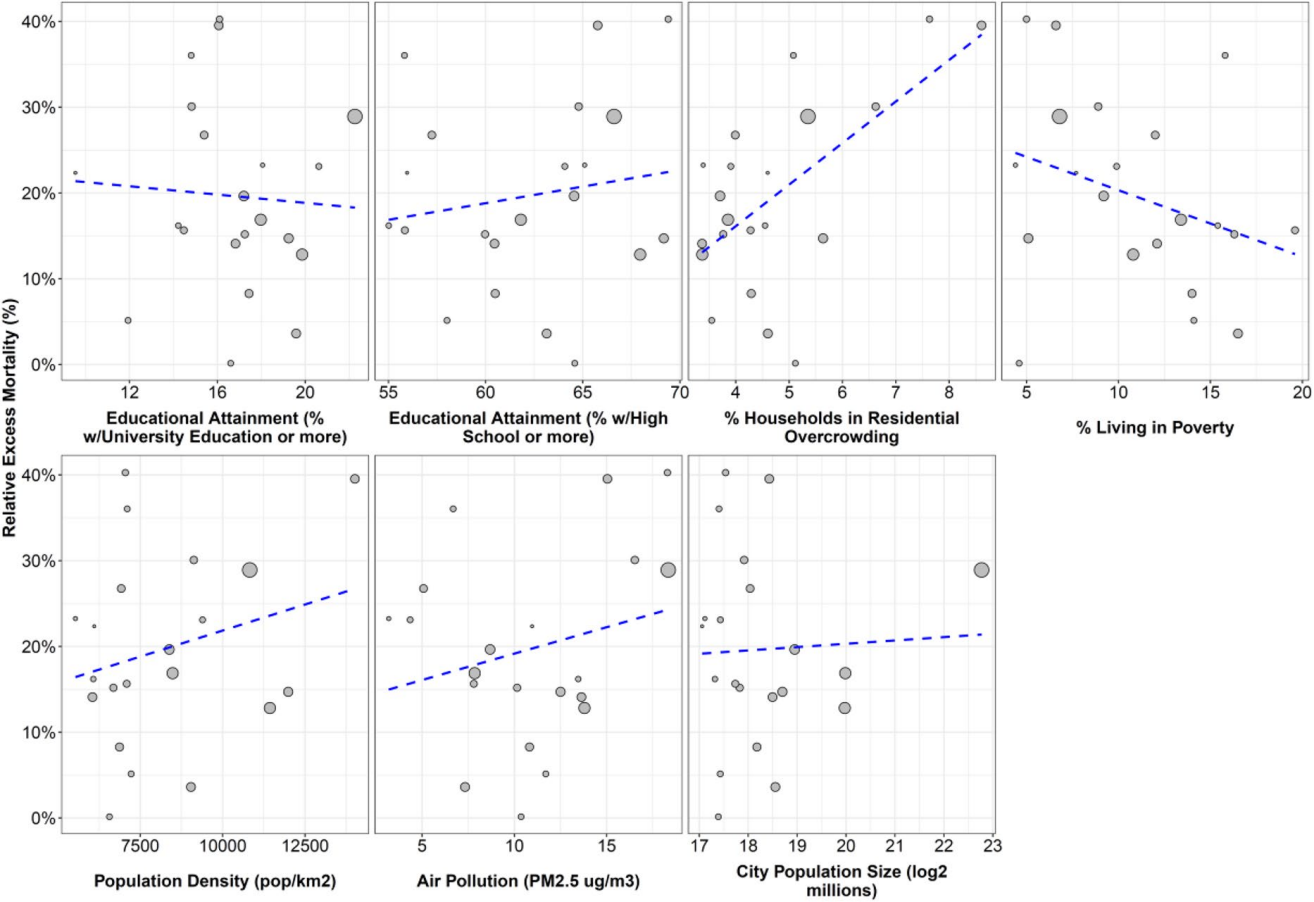
Correlation between excess mortality during pandemic (2020 to June 2021) and selected urban factors in 21 cities of Chile

**Fig. 5:**
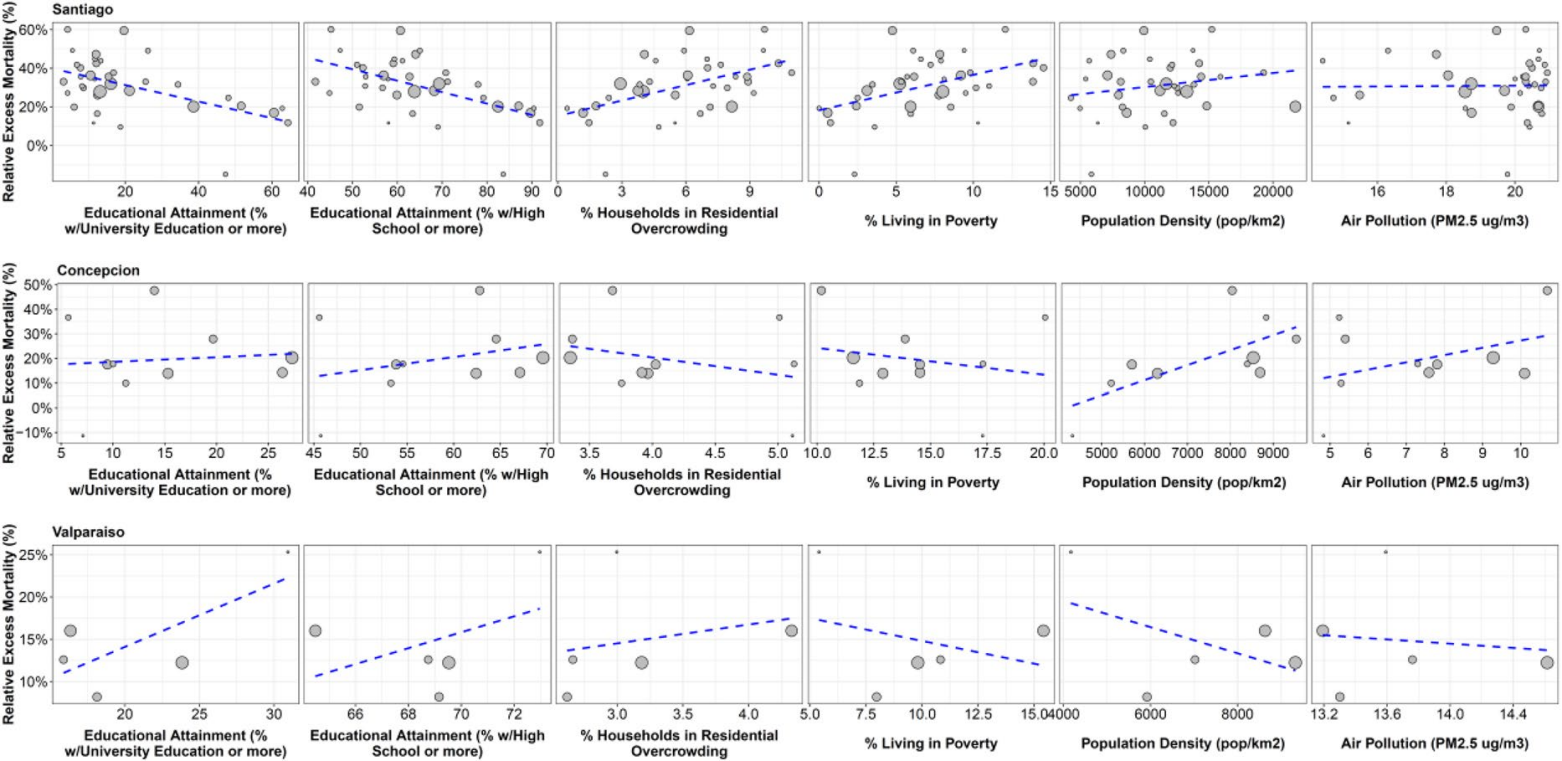
Correlation between excess mortality in 2020-June 2021 and selected urban factors in the municipalities of three Metropolitan Areas of Chile

Figures 4 and 5 show the associations between social and built environment urban factors and relative EM, first for all cities and then for municipalities in the three largest metropolitan areas in Chile (Santiago, Valparaíso and Concepción), while Table 2 shows the strength of these associations, in absolute and relative terms. At the city-level, we found that cities with higher residential overcrowding had higher EM: a 1-SD increase in city-level overcrowding was associated with 8.1% higher EM (95%CI 2.4 to 13.8%) and 38.99 higher deaths per 100,000 (95%CI 10.02 to 67.96). We found no associations between city-level relative or absolute EM and educational attainment, poverty, population density, air pollution, or city population size. Supplementary material Figs. 10 and 11 show the result of the sensitivity analysis using bootstrapping to acknowledge uncertainty around the estimates of EM, with no changes in point estimates and a widening of CI that does not affect our main inferences. We found a few minor differences in the analysis but municipality, but these should be interpreted with caution as we were unable to obtain bootstrapped estimates of EM for one municipality of Concepción and Valparaíso each.

**Table 2:** Association between urban factors and excess mortality for 21 cities in Chile and municipalities of three metropolitan areas

Variable	SD	Relative Excess Mortality (95% CI)	Absolute Excess Mortality (95% CI)
City-level (*n* = 21)			
Educational Attainment (University)	2.9%	–1.62 (–6.52;3.28)	–8.08 (–32.63;16.48)
Educational Attainment (High School)	4.6%	–0.02 (–5.47;5.43)	–1.48 (–28.78;25.83)
Residential Overcrowding	1.4%	8.09 (2.4;13.78)	38.99 (10.02;67.96)
Poverty	4.5%	–2.35 (–7.51;2.82)	–13.76 (–39.43;11.91)
Population Density	2262 pop/km2	1.59 (–3.52;6.69)	6.2 (–19.5;31.9)
Air Pollution	4.3 ug/m3	1.73 (–3.23;6.7)	5.45 (–19.64;30.54)
City Population Size	Doubling	0.45 (–4.39;5.28)	0.63 (–23.64;24.89)
Municipality-level			
Santiago (*n* = 36)			
Educational Attainment (University)	17.9%	–7.23 (–11.73;-2.73)	–62.12 (-89.24;–35.01)
Educational Attainment (High School)	13.6%	–7.64 (–11.92;-3.36)	–65.77 (–90.76;–40.77)
Residential Overcrowding	2.8%	7.04 (2.68;11.4)	62.68 (37.04;88.33)
Poverty	3.8%	6.4 (1.73;11.07)	59.96 (32.07;87.85)
Population Density	3974 pop/km2	3.11 (–1.7;7.92)	32.03 (0.86;63.2)
Air Pollution	1.9 ug/m3	2.84 (–2.84;8.52)	26.93 (–10.59;64.45)
Concepción (*n* = 10)			
Educational Attainment (University)	7.6%	3.02 (–8.99;15.03)	12.54 (–52.2;77.28)
Educational Attainment (High School)	8.6%	5.75 (–5.27;16.77)	24.49 (–36.11;85.08)
Residential Overcrowding	0.7%	–4.94 (–15.8;5.92)	–19.17 (–78.87;40.54)
Poverty	3.0%	–2.89 (–14.26;8.47)	–4.12 (–65.97;57.73)
Population Density	1803 pop/km2	11.44 (3.64;19.24)	61.54 (20.02;103.05)
Air Pollution	2.1 ug/m3	6.52 (–3.89;16.93)	31.98 (–24.68;88.65)
Valparaíso (*n* = 5)			
Educational Attainment (University)	6.4%	2.92 (0.28;5.57)	7.19 (–19.86;34.24)
Educational Attainment (High School)	3.0%	1.09 (–3.32;5.49)	–6.12 (–32.54;20.3)
Residential Overcrowding	0.7%	1.11 (–3.01;5.24)	15.93 (1.76;30.11)
Poverty	3.7%	–0.88 (–5.19;3.44)	6.76 (–18.28;31.8)
Population Density	2078 pop/km2	0.18 (–5.25;5.62)	11.46 (–16.81;39.72)
Air Pollution	0.6 ug/m3	1.2 (–3.12;5.52)	1.63 (–25.85;29.11)

Footnote: results come from a linear model of relative excess mortality on city- or municipality-level factors, adjusted by age. Relative excess mortality was defined as excess mortality over the number of expected deaths (obtained from the GAM model). Absolute excess mortality was defined as the difference between observed deaths and expected deaths divided over the population. CI means confidence intervals

We found a different pattern in the three metropolitan areas. First, municipalities within Santiago with higher educational attainment had lower EM (-7.2% and -7.6%, 95%CI –11.7 to –2.7 and -11.9 to -3.4, per 1-SD increase in educational attainment at the university and high school levels, respectively), while municipalities with higher overcrowding and poverty had higher EM (7.0, 95%CI 2.7 to 11.4, per 1-SD increase in overcrowding and 6.4%, 95%CI 1.7 to 11.1 per 1-SD increase in poverty). These associations also existed with absolute EM. We found an association between population density and relative and absolute EM in Concepción. Last, we found an association between education and relative EM, and between overcrowding and absolute EM in Valparaíso, but these associations were inconsistent in the absolute and relative scales.

## Discussion

In this study, we found that all-cause mortality in Chile was 13% and 11% higher during 2020 and 2021 (up to July 3^rd^), as compared to the 2016–2019 period, resulting in around 22,000 total excess deaths from January 1st, 2020 to July 3^rd^, 2021. This increase was more marked among men and among the 20–39 years age group and older adults (60 to 74 and older than 75 years old). We found a heterogeneous impact between and within cities, and over time, with the Santiago Metropolitan region being the most affected region in 2020 (especially during June and July, 2020), and other regions in the following months (December 2020 to June 2021). Last, we found that cities with higher levels of overcrowding had higher EM, while areas of Santiago with higher overcrowding and lower educational attainment had higher EM, as did areas of Concepción with higher population density.

Our results are consistent with other Latin-American studies, reporting higher EM in men, adults and older adults and during the winter in the first wave [42, 43], as well as in capital cities [43, 44]. We found a 13% relative EM for 2020, consistent with the nationwide estimations of the Ministry of Health for 2020 (13%) [34], and 11% during 2021. EM has been reported since the beginning of the pandemic in North American and European countries [10, 24, 45], and more recently in Latin America, with high variability in the estimations [46]. México reported an EM of 42.8% at the end of 2020 [44, 47] and 45% by June 2021 [47]. In Perú, a nationwide 139.8% EM was reported between April-June 2020 [48], while in Brazil a 45% EM was observed between February and June 2020 in four metropolitan regions, varying between 112% in Manaus to 34% in Sao Paulo [49]. In Guatemala, a nationwide peak of EM was reported during week 28 (July) 2020 up to 73% of EM [2], while in Perú, a peak of 189.6% was reported in week 32 [50]. At city-level, in Brazil there was reported a peak of EM in Manaus during epidemiological weeks 17–20 up to 296% [49]. In our study we found a peak in EM (above 140%) in Santiago during weeks 23 and 24 of 2020, consistent with a peak of 77% and 75% during the same weeks in all 21 cities pooled, coincident with higher COVID-19 transmission observed in the northern hemisphere.

We found a heterogeneous impact of COVID-19 between and within Chilean cities, with the highest increases in mortality in three cities of the North of Chile, and wide variations within the metropolitan areas of Santiago and Concepción. Increases in mortality varied over time according to macroregion, peaking first in Santiago and followed by the North region. The early peak of Santiago may be related to its role as the country’s Capital, with people travelling from outside Chile, explained by an earlier introduction of cases [51] at a time where knowledge about the control of transmission was limited and mitigation measures had not yet been implemented. The north of Chile is a mining zone, one of the principal extractive activities of the country, where lockdowns arrived at a later stage than in other regions in the country [52, 53], with important variations in their mobility [54], probably given the centrality of their economic activity [55], explaining a higher COVID-19 incidence in Calama and Antofagasta, and, potentially, higher relative EM. Consistently, the occupation of hospital beds in the North was reported at the limit of its capacity many times during 2020 [56]. The region of Tarapacá in the North, showed important increases in the percentage of poverty and extreme poverty during pandemic [57], which could be revealing precarious jobs and worse access to health care services and their consequences in mortality. Particularly in Tarapacá, Alto Hospicio, the municipality which the highest EM in the whole period, is an area characterized by informal settlements, high immigration and poverty [58–60]. EM started increasing substantially during the Summer of 2021 in the Center and South regions too, a phenomenon has been attributed to mobility during the summer vacations, where residents of Santiago travelled to other locations [61]. In fact, EM mimics the trends in incidence during the pandemic. This may change in the coming months if there is a surge in mortality now not due directly to COVID-19, but indirectly given the delay in diagnosis and treatment of other potentially severe conditions such as cancer and cardiovascular disease [62].

The variations in EM across different countries and cities have been attributed to different factors, including variability in COVID-19 fatality rates, mortality due to delays in health care for other conditions, and reduction in mortality due to injuries and respiratory viruses [3]. Variation due to social and environmental conditions has been described too [48, 49], especially in countries where cities concentrate worse socioeconomic conditions [63]. Chile is a country with very wide inequalities [64], including health inequalities [28, 65]. We found an association between EM and overcrowding at both the city and municipality levels, while we found that municipalities of Santiago with lower educational attainment had higher EM, consistent with previous reports [23, 30]. Previous studies shown a similar relation at the county level, considering the association between overcrowding and COVID mortality [63, 66, 67]. Ahmad et al. reported a 42% higher mortality risk by COVID for every 5% of increase in poor housing conditions in USA counties at the beginning of the pandemic [67]. Lower EM was associated with higher Human Development Index (HDI) in the Metropolitan Area of Lima, Perú [42]. In Chile, COVID-19 studies done in Santiago, showed consistent results with ours. Mena et al. found a strong association between socioeconomic factors and incidence, mortality and testing capacity of COVID-19 in Santiago [23], while Gozzi et al. reported different impact of the non-pharmaceutical interventions according to the HDI of the municipalities of Santiago [30]. These associations between social factors and EM could be originated by differential exposure to SARS-CoV-2, due to the differential capacity to adhere to mitigation measures for those with precarious jobs and residential overcrowding, among others. Differential vulnerability to severe COVID-19 may also be behind these findings, as there are differences in access to healthcare in Chile, depending on private vs public provision, as public provision concentrates poorest and sickest population [68], along with a high prevalence of risk factors for those of lower socioeconomic status [68, 69].

We found different associations in the metropolitan areas of Concepción and Valparaíso. First, in Valparaíso we found inconsistent associations between educational attainment and overcrowding with relative and excess EM, while we found no associations for the same indicators in Concepción. These differences with Santiago could be due to three potential reasons. First, the low sample sizes (*n* = 10 and 5 municipalities in Concepción and Valparaíso, respectively, compared to the 36 of Santiago). Second, educational attainment (and overcrowding, to a degree) are measures of socioeconomic status [70]. If both social inequalities and economic residential segregation are wider in Santiago, which is one of the large Latin American cities with widest health inequalities [28], as compared to Concepción and Valparaíso, associations between these indicators and EM will be weaker in these two metropolitan areas. Third, there may be differences in baseline mortality across these cities. We explored how they were associated with EM (supplementary material Figs. 4 and 5), finding that relative (but not absolute) EM was negatively associated with baseline mortality. This means that relative EM is sensitive, as expected, to baseline levels of mortality. To address this, we explored the association of social and environmental factors with two measures of EM (relative and absolute), but these findings were also inconsistent in Concepción and Valparaíso.

Last, we explored the association between built environment factors (population density and air pollution) and EM. We did not find any association, except for population density in Concepción. Population density have been reported previously as potential factor driving EM [71] at the county level, but other studies have not found the same association [63]. Studies in Northern Italy, Germany and USA [39, 72, 73] found associations suggesting increases in COVID-19 related mortality per one-unit increase in PM2.5 concentration (μg/m3), but other studies have not found association between mortality and air pollution at the municipality level, where demographic and socioeconomical factors may be stronger drivers of EM^74^.

### Limitations

We acknowledge some limitations. There may be delays in registration, although in Chile, deaths should be registered up to three days according to the law. Regardless, we allowed for up to 2 months in delays by using data on deaths registered up to September 1^st^ 2021, to cover the period of deaths in study. Due to the dynamics of the pandemic in Chile, we examined EM up to June 2021 to account variability in the different regions of Chile, but it implies that we include the possible effect on mortality of the vaccines, since the vaccination campaign started on February 2021, reaching coverages over 70% at the beginning of July. If the distribution of these vaccines occurred unevenly, especially by the factors we examined in this study, some of our results for the first half of 2021 may be related to these patterns in vaccine rollout. There are risk factors and indicators of health access that could have affected EM but were outside the scope of this study. Future work would consider the role these play as mediators in the pathway between socioeconomic status (proxied by educational attainment) and mortality. Relatedly, and given the limited number of municipalities within each city, we had a limited capacity to examine within-city inequalities outside of Santiago. Despite that, our results highlight the situation of other cities than the capital, less explored in our country by now. Last, our PM2.5 data relies on satellite imagery and corresponds to 2018, and may represent and incomplete picture of air quality conditions in these cities.

## Conclusion

We found a heterogeneous increase of mortality in urban areas of Chile during the COVID-19 pandemic, with the capital and Northern cities being most impacted with relative EM of upwards of 20% from January 2020 to June 2021, and peaks of up to 150% in the beginning of winter 2020 in Santiago. We showed that EM has been higher during 2021 than 2020 outside the Metropolitan region, suggesting the importance to continue analyzing the geographic heterogeneity in EM during the pandemic. EM was higher in cities with higher levels of housing overcrowding and, within Santiago, in municipalities with higher overcrowding and lower educational attainment, indicating the impact of social inequalities on health outcomes in Chile. Continuing the monitoring of EM is crucial to evaluate the effects of the pandemic in the following years. Furthermore, a geographically and socially disaggregated reporting of EM is useful to understand the entire impact of the pandemic, allowing for the prioritization of specific population groups and territories in interventions such as vaccination campaigns.

## Supplementary Information

Below is the link to the electronic supplementary material.

Supplementary file1 (DOCX 1.26 MB)
