# Supplementary material for: Excess Mortality during the COVID-19 Pandemic in Cities of Chile: Magnitude, Inequalities, and Urban Determinants
Source: J Urban Health. 2022 Jun 10;99(5):922–35. doi: 10.1007/s11524-022-00658-y (PMC9187147; doi:10.1007/s11524-022-00658-y)
Supplement: Supplementary file 1 — Supplementary file1 (DOCX 1.26 MB) [file 11524_2022_658_MOESM1_ESM.docx]

**Supplementary Material 1: Ancillary results**

**Fig. 1: Mortality rate in Chile before and during pandemic, 2016-June 2021, all of Chile and in cities bigger than 100.000 residents**

**Fig. 2: Mortality rate in Chile before and during pandemic, 2016-June 2021, by sex**

**Fig. 3: Mortality rate in Chile before and during pandemic, 2016-June 2021, by age:**

**Fig.4: Mortality rate in Chile during pandemic, 2016-June 2021, by city:**

**Fig. 5: Comparison between mortality rate, relative and absolute excess mortality in Chile during pandemic, 2016-June 2021, SALURBAL cities**


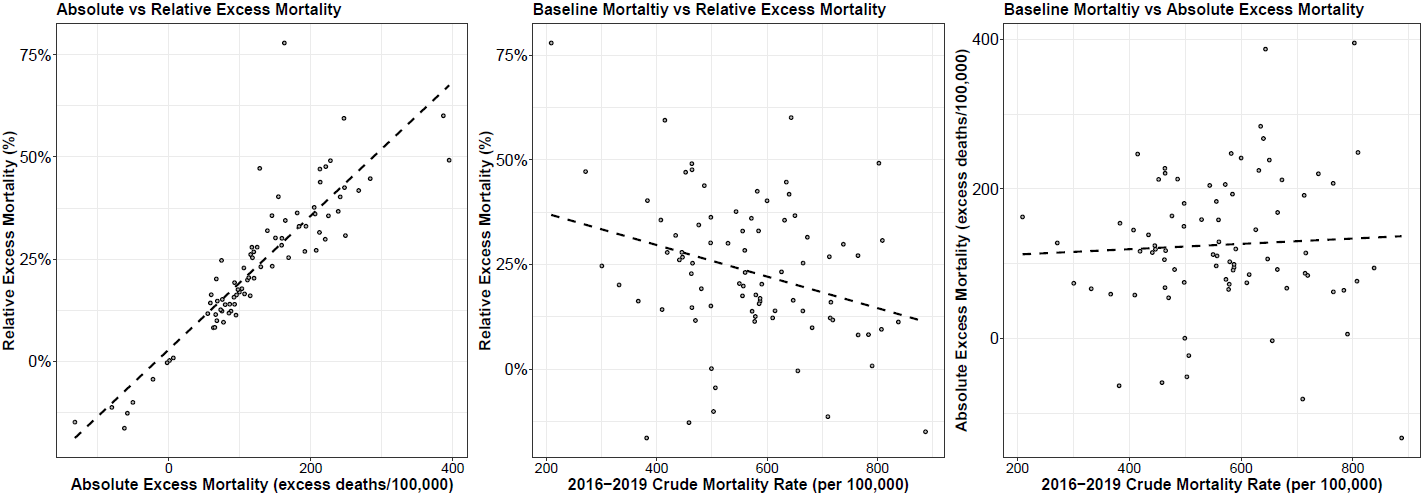


**Fig. 6: Excess Mortality from January 2020 to June 2021 in 21 Chilean cities included in this study**

Footnote: area of circle is proportional to the total number of excess deaths. Fill of circle refers to the relative excess mortality (%) or excess mortality rate per 100,000.

**Fig. 7: Comparison of expected and observed counts using three different baseline periods starting in 2016, 2017, and 2018.**

Footnote: vertical solid line represents first week of observations and vertical dashed line represents last week of observations used in model.

**Fig. 8: Comparison of relative and absolute excess mortality between the main analysis (baseline period in 2016-2019) and two different baseline periods (2017-2019 and 2018-2019) by city in 21 Chilean cities.**

Footnote: (A and B) represents relative excess mortality, (C and D) represents absolute excess mortality (excess mortality rate); (A and C) compare 2016-2019 to 2017-2019; (B and D) compare 2016-2019 to 2018-2019.

**Fig. 9: Comparison of relative and absolute excess mortality between the main analysis (generalized additive model) and an empirical comparison with weekly average deaths in 2016-2019 by city in 21 Chilean cities.**

Footnote: (A) represents relative excess mortality, (B)represents absolute excess mortality (excess mortality rate).

**Fig. 10: Comparison of effect estimates and confidence intervals in the main analysis vs bootstrap approach, at the city level**

Footnote: (A) shows associations between each exposure and relative excess mortality, and (B) shows associations between each exposure and absolute excess mortality rates.

**Fig. 11: Comparison of effect estimates and confidence intervals in the main analysis vs bootstrap approach, at the municipality level**

Footnote: (A) shows associations between each exposure and relative excess mortality, and (B) shows associations between each exposure and absolute excess mortality rates. Note that analysis in Concepción and Valparaíso use one fewer municipality compared to the main analysis, as we were unable to obtain bootstrapped estimates of excess mortality in two small municipalities.

**Supplementary Material 2: model used to estimate excess mortality.**

Estimation of Excess Mortality

We estimated weekly and total (sum for the whole period) excess mortality (EM) and associated 95% confidence intervals by cities pooled in macroregions (North, Center, Metropolitan, South), each city, and each municipality. EM is defined as the difference between actually observed deaths and a counterfactual that is assumed to be the death count had no discrete event (in this case the COVID-19 pandemic) occurred. Given that mortality in Chile shows strong seasonal behaviors, with peaks in the winter (June to September), we need a model that accounts for this seasonality. To estimate this counterfactual while accounting for seasonality, we computed a smoothed estimate of expected death counts for each week of 2020 and up to June 2021 based on data from 2016 to 2019 and using a negative binomial generalized additive model (GAM). By comparing the same epidemiological week to prior years, seasonality can be accounted for. This approach is detailed in more detail in Basellini et al. ^1^, but a summary follows.

For a specific city, we have a matrix of deaths $\boldsymbol{D}=(d_{wy})$ where w indexes the epidemiological week and y indexes the year. We assume that each item $d_{wy}$ of this matrix follows a negative binomial distribution, given the usual overdispersion of mortality counts^1^. Then, considering $\mu_{wy}=E(D_{wy})$ as the expected value of deaths under the negative binomial process, we model this number of deaths using a generalized additive model (GAM) of the form:

$$\log\left( \mu_{wy} \right)=\log\left( pop_{wy} \right)+f\left( x_{wy} \right)+\alpha_{0}+\alpha_{w}$$

Where $pop_{wt}$ is the offset, or population denominator, which we assume to be constant over the year (but varying by year), and that we obtained from population projections (see main paper). $\alpha_{0}$ is the intercept and $\alpha_{w}$ is a week-specific intercept that captures time-varying but spatially-fixed changes, with the first week being the reference group to ensure identifiability of the model^1^. This week-specific effect controls for seasonality. Last, the function $f\left( x_{wy} \right)$ is a smooth function over the entire set of observations $(x_{wy}=1, \ldots, m)$ where m is the total number of observations. This function captures long-term trends in mortality.

We then used this model to predict weekly expected death counts and standard errors during 2020 up to June 2021. We then calculated absolute excess mortality as defined as the difference between observed deaths and expected deaths, divided over the population in 2020 and 2021. Relative excess mortality was defined as the difference between observed deaths and expected deaths. To calculate excess mortality for the entire period, we summed over the total number of expected and observed deaths from January 2020 up to June 2021, and calculated excess mortality using the same definitions as above.

Sensitivity Analyses

We conducted two sensitivity analyses to assess the robustness of our excess mortality estimates. We tested different three baseline windows for our GAM model, starting in 2016 (main analysis), 2017 and 2018, and in all cases going up to 2019. Second, we followed Basellini et al^1^, and compared weekly average of deaths during the 2016-2019 period and compared that with deaths in the same week of 2020 and 2021. This is termed empirical estimate in the main manuscript.

Associations between municipality and city-level factors and excess mortality

In the second part our paper, we explore potential predictors of excess mortality both at the city level and the municipality level (for the three largest metropolitan areas). For this, we compute cumulative excess mortality by week 26 of 2021 by summing over expected and observed deaths during 2020 and 2021 (see above for details on this). We then calculated both absolute excess mortality (excess mortality rate and relative excess mortality). To estimate associations between factors and absolute and excess mortality, we used a linear model of the form:

$$EM_{c}=\beta_{o}+\beta_{1}*\%Poverty_{c}+\beta_{2}*\%Age{65}_{c}+\epsilon_{c}$$

Where EM_c_ represents excess mortality in each city or municipality indexed by c, in its absolute or relative form, $\beta_{0}$ is an intercept, $\beta_{1}$represents the change in excess mortality per 1-standard deviation increase in % poverty, and $\beta_{2}$ is an adjustment coefficient for the proportion of the city or municipality that is aged 65 or above; last, $\epsilon_{c}$ is the error term.

To acknowledge uncertainty around the estimates of excess mortality, we conducted a secondary analysis employing the bootstrapping approach described in Basellini et al^1^. In summary, we employed a bootstrap approach to simulate 1000 death counts for each week/unit combination from the deviance residuals of the GAM. For each of these simulations, we repeated our estimation of EM, resulting in 1000 estimates of EM from January 2020 to June 2021 per city or municipality. We then fit 1,000 linear models and pooled point estimates and standard errors of coefficients using Rubin’s formula^2^.We conducted this analysis as a secondary analysis as we were unable to obtain bootstrapped estimates for two small municipalities in Concepción and Valparaíso.

**References**

1. Basellini U, Alburez-Gutierrez D, Del Fava E, Perrotta D, Bonetti M, Camarda CG, Zagheni E. Linking excess mortality to mobility data during the first wave of COVID-19 in England and Wales. *SSM - Population Health* 2021;**14**:100799.

2. Rubin DB. *Multiple imputation for nonresponse in surveys*. Vol. 81 John Wiley & Sons, 2004.
